# Interactions between the Gut Microbiome, Lung Conditions, and Coronary Heart Disease and How Probiotics Affect These

**DOI:** 10.3390/ijms22189700

**Published:** 2021-09-08

**Authors:** Trudy M. Wassenaar, Valentina A. Juncos, Kurt Zimmermann

**Affiliations:** 1 Molecular Microbiology and Genomics Consultants, Tannenstrasse 7, 55576 Zotzenheim, Germany; 2 Department of Biomedical Informatics, University of Arkansas for Medical Sciences, Little Rock, AR 72209, USA; vajuncos@uams.edu; 3 Symbiopharm GmbH, 35745 Herborn, Germany; kurt.zimmermann@symbio.de

**Keywords:** gut–lung axis, gut microbiome, coronary heart disease, COVID-19, probiotics, short-chain fatty acids

## Abstract

The importance of a healthy microbiome cannot be overemphasized. Disturbances in its composition can lead to a variety of symptoms that can extend to other organs. Likewise, acute or chronic conditions in other organs can affect the composition and physiology of the gut microbiome. Here, we discuss interorgan communication along the gut–lung axis, as well as interactions between lung and coronary heart diseases and between cardiovascular disease and the gut microbiome. This triangle of organs, which also affects the clinical outcome of COVID-19 infections, is connected by means of numerous receptors and effectors, including immune cells and immune-modulating factors such as short chain fatty acids (SCFA) and trimethlamine–N–oxide (TMAO). The gut microbiome plays an important role in each of these, thus affecting the health of the lungs and the heart, and this interplay occurs in both directions. The gut microbiome can be influenced by the oral uptake of probiotics. With an improved understanding of the mechanisms responsible for interorgan communication, we can start to define what requirements an ‘ideal’ probiotic should have and its role in this triangle.

## 1. Introduction

The gut microbiome is a crucial factor for the overall health of an individual, but its relationship with a wide variety of medical and psychosomatic conditions is complex. The variation in microbiome constituents between healthy individuals is extensive [1]. This interindividual variation is the result of many factors including diet, genetics, exposure to microbial communities and infections, and medication [2,3]. Despite the variation in microbiome makeup, common patterns can be recognized, which roughly correlate with the health status of individuals and a range of disease states. The gut microbiome can become distorted by a large number of conditions and their treatments, but microbiome distortions can also contribute to the onset or severity of such conditions, so that it is sometimes difficult to unravel cause and effect. The gut microbiome is in constant direct communication with the immune and neuroendocrine systems and also exchanges information indirectly with the brain via the vagus nerve, in addition to supplying nutrients and other ingested compounds to the bloodstream. As such, it is a key player involved in both surveillance and mitigation of general physiology and health. Except for temporal distortions, the individual gut microbiome is relatively stable from around the age of three years onwards [4]. Thus, throughout life the metabolic capacity of an individual’s gut microbiome remains fairly constant, as long as diet and health status do not change. However, its composition can be affected by pathogens (bacterial, fungal, viral, and parasitic), drugs with intentional or accidental antimicrobial activity, or by intake of nonpathogenic or probiotic organisms.

It is becoming increasingly clear that the gut microbiome has a major impact on cardiovascular health. Coronary atherosclerosis and related myocardial impairment are the major contributors to morbidity and mortality in the Western world. Recognized risk factors for cardiovascular diseases include hypertension, diabetes mellitus, smoking, and adverse lifestyle. Here, we use the term cardiovascular diseases (CVD) to collectively describe atherosclerotic coronary disease and its consequences such as heart failure and thrombosis. Adverse lifestyle includes dietary patterns, which provide a potential target for treatment and prevention. The gut microbiome has been shown to differ in CVD patients and in CVD-at-risk populations compared to controls, although cause and effect cannot always be separated. Most likely, the gut microbiome and the host’s health status reciprocally affect each other in multiple and complex interactive ways.

The interplay between the gut microbiome and lung health has been known for some time but has strongly resurfaced during the COVID-19 pandemic [5,6]. The SARS-CoV-2 virus primarily infects the upper airways but has strong complications in the lungs. In a number of mild to severe COVID-19 patients, the gut microbiome is severely disturbed, and this disturbance can be sustained over a period of months. As with other respiratory viral infections, COVID-19 can be accompanied by gastrointestinal symptoms and the viral RNA can be detected for prolonged periods following the end of symptoms in the stool of infected individuals [7]. Disturbance of the gut microbiome as a result of airway infections does not necessarily depend on the virus actively replicating in the gut, as routes of gut influence exist via the immune and nervous systems, which not only react towards the gut microbiome but also regulate its composition. Immune regulation works in both directions, affecting both the lung and gut microbiomes. A healthy gut microbiome composition can contribute to a higher resistance to airway infections and their symptoms, as has been demonstrated for common cold infections.

A link between CVD and COVID-19 (and other viral lung infections) is also clear, as both blood clotting and infection of cardiac tissue are common complications of COVID-19, both strongly contributing to its mortality. The SARS-CoV-2 infection indirectly results in endothelial cell damage of the capillaries that contribute to the micro-thrombosis that causes multiple organ damage that is the hallmark of severe COVID-19 [8]. Risk factors for CVD, such as obesity or diabetes, are also risk factors for increasing the severity and mortality of COVID-19. As will be discussed here, the gut microbiome regulates responses involving the immune system, cardiovascular disease, and lung health, for which the mechanisms are now better understood. This opens the possibility to target the health of the gut microbiome with probiotics, as it is becoming clear which functions are mainly responsible for their health benefits.

## 2. The Gut Microbiome and CVD

It is well established that there is a relationship between the gut microbiome and CVD. The most detailed insights on the human gut microbiome stem from metagenomic investigations. By deep-sequencing or shotgun metagenomics the sequence of all microbial DNA in a given sample is determined. Metagenomic (shotgun) sequencing has been used to define a normal (healthy) gut microbiome [9]. Such work has illustrated that there is a vast variation among individuals, even though the method suffers from a lack of sequencing depth of the bacterial community, so that low abundant species are likely to be missed. The human microbiome is more often studied by sequencing a 16S rRNA gene fragment amplified by PCR using ‘universal’ primers. The sequences from these short 16S fragments reveal far less organism information than shotgun sequencing, so the precise species often cannot be determined.

A number of investigations have determined the microbiome sequences of stools from CVD patients, often by comparing CVD patients to healthy controls. Considerable variation between patient populations, as well as outcomes, exist in the available literature, but in broad strokes, in the absence of disease, the human gut is dominated by members of the Bacteroidetes phylum, followed by Firmicutes, while Proteobacteria and Actinobacteria are present at much lower numbers. A bacterial phylum may represent hundreds of species, some of which can be found in the human gut, that have a wide variety of ‘microbiome functions’. Even within a species (at the other end of the spectrum of precision in describing a microbiome), individual strains can still vary considerably in their properties. For example, the genomes of strains of *Escherichia coli* can vary in size by millions of basepairs, and the species harbors both commensal and pathogenic strains. Thus, even if the dominant phyla are identified, the actual species and their functional characteristics can vary considerably between individuals.

When comparing CVD cases to healthy controls, most of the metagenomic studies have reported an increased abundance of Firmicutes and Bacteroidetes, and decreased numbers of *Roseburia* (*R. intestinalis*) or *Faecalibacterium* (*F. prausnitzii*) in CVD cases, although 16S-dependent studies did not always report these differences [10]. Further, within the Firmicutes, often Lactobacillales, including *Lactobacillus*, *Enterococcus*, and *Streptococcus* species are often more abundant in CVD patients.

A reduction in microbiome species diversity is considered a health risk and reduced species richness was found to be associated with CVD disease. A decreased abundance in at least some members of the phylum Bacteroides is frequently observed in CVD patients, in particular in patients at risk with obesity, diabetes, and hypertension. A higher abundance of Gram-negative pathogens is sometimes observed in CVD patients.

Two major mechanisms for how the gut microbiome may be involved in CVD have been proposed; these are not mutually exclusive. The first depends on the formation of trimethylamine–N–oxide (TMAO) and the second on the production of short-chain fatty acids (SCFA).

TMAO is a constituent of certain foods (such as red meat, eggs, dairy products, and marine fish). About three-quarters of consumed TMAO is secreted in the urine within 24 h [11], while approximately one-third is reduced in the gut to trimethylamine (TMA) by bacterial TMAO reductase [12]. TMA is a fishy-smelling gas that is rapidly adsorbed and oxidized by the liver to TMAO by monooxygenase 3 (FMO3). TMA can also be produced by gut bacteria from the utilization of dietary quaternary amines such as phosphatidylcholine (lecithin), choline, and L-carnitine. The major source of TMAO in the body is TMA produced by gut bacteria from food-derived choline and carnitine.

A clear correlation exists between plasma TMAO levels and the risk of CVD or thrombotic disease [13,14]. TMAO levels also correlate with obesity [14]. A meta-analysis of 19 studies concluded that elevated TMAO levels are associated with increased risk of major adverse CVD events [15]. Of all the mechanistic explanations of how the gut microbiome may affect heart conditions, the action of TMAO has been described as “the first potentially direct link between the gut microbiota and atherosclerotic heart disease” [16].

The other well-recognized mechanistic link between the gut microbiome and CVD is the production of SCFA, which are formed when bacteria ferment dietary fibers. In particular, the SCFA butyrate is considered to contribute to a healthy gut, as it stimulates gut epithelial integrity. When the epithelium of the gut is damaged, bacterial lipopolysaccharide (LPS, also called endotoxin) can leak out and enter the bloodstream (endotoxemia), where it has potent pro-inflammatory activity. Temporary endotoxemia is normal following a high-fat meal, which results when LPS is cotransported together with fats across the gut’s membranes [17]. Such LPS is quickly removed from the bloodstream by specific binding proteins that transport it to the liver. However, chronic endotoxemia, as a result of what is somewhat inaccurately called a ‘leaky gut’, results in a permanent stage of low-grade inflammation as a result of endotoxemia, with negative effects on general health, including an increased risk of type-2 diabetes and CVD [18,19]. Butyrate produced by ‘good’ gut bacteria assists in the regeneration of the gut epithelium barrier, reducing LPS leakage [20].

A low-fiber diet results in microbial dysbiosis and reduced bacterial SCFA production, conditions that can lead to a chronic inflammatory and endotoxemic state, which are associated with CVD. A high-fat diet can also be unhealthy, with lipemia and obesity also linked to endotoxemia. Moreover, low fiber intake and high sugar intake are associated with increased glycemia and often with obesity or diabetes, which are risk factors of CVD. That gut bacteria can ameliorate a diabetic state has been shown by fecal transplants: when gut microbiota obtained from a nondiabetic donor is transplanted to a diabetic individual, it improves glucose metabolism, at least temporarily, as was confirmed in a systematic review [21].

SCFAs are mainly produced in the colon by bacteria that degrade carbohydrates, abundant in dietary fiber that cannot be digested by the host [20]. The general health-promoting effect of dietary fibers is widely accepted and supported by meta-analyses [22]. The colonic bacteria produce a mixture of SCFA, with approximately 15% butyrate [20], resulting in a weakly acidic pH of the colonic lumen. Butyrate has become the focus of attention, as it is produced by common gut bacteria [20,23], and it stimulates gut epithelial integrity by stimulating the repair of any local damage of the intestinal lining [20,23]. A role for butyrate-producing bacteria in gut integrity also fits with the observations that inflammatory bowel disease (IBD), a chronic condition commonly accompanied with dysbiosis, is associated with low SCFA/butyrate production in the gut and can be improved with SCFA supplementation [20]. Indeed, switching from a high-fiber, low-fat diet to a low-fiber, high-fat diet results in an increase in serum inflammatory markers, and a low-fiber diet results in lowered butyrate levels in the gut [24]. A number of common gut bacterial species are able to produce butyrate, of which *Faecalibacterium prausnitzii* and *Eubacterium rectale* (both Clostridia members, Firmicutes) are quantitatively most important. Each of these can make up 5 to 10% of a healthy gut microbiome [25]. Differences in the abundance of these and other Firmicutes that can potentially produce butyrate have been shown between CVD patients and controls. The intestinal bacterial community forms an intricate metabolic network, so that abundance of one functional group can affect that of other groups. Thus, the presence of butyrate-producing bacteria can be influenced by manipulating the abundance of others, such as *Bifidobacteria*, as was shown in mixed culture fermentation experiments [26,27].

To complicate the interplay between heart and gut, cardiac dysfunction can damage gut functions and cause a leaky gut, resulting in endotoxemia that then negatively affects the heart again. Gut ischemia can develop as a result of elevated gut venous pressure and decreased blood flow [28]. The resulting changes in gut physiology (e.g., sodium secretion and electrolyte absorption) can then affect the function and composition of the gut microbiome, and with such feedback loops in place, it can be difficult to unravel cause and effect, but an altered gut microbiome frequently accompanies chronic heart failure.

## 3. The Gut Microbiome and Lung Health

During embryonic development, both the gut and the lungs are formed from the same primitive foregut. After birth, both organs function as a mucosal barrier between deeper tissue and the external environment, and they must both keep local microbial populations in check; at the same time, they must allow the passage of essential nutrients (the gut) and gases (the lungs) [29]. The developing fetus is already potentially exposed to a microbiome, although this has been debated [30]. Regardless, immediately after birth, both the lungs and the intestines are colonized with an early microbiome, which, in terms of dominant bacterial phyla, in both organs resemble each other at that stage [29,31].

From a certain age onwards, in a healthy state, the lungs are home to a specific microbiome that is dominated by the bacterial phyla Bacteroidetes and Firmicutes, just like the gut microbiome, although the dominant genera and species may differ between these organs. However, the gut contains at least a million more bacterial cells per gram tissue than the lungs do [29]. These organs sense and communicate with their normal microbiome, and the mechanism behind this host–microbe interaction, how the gut and lungs respond to pathogens, and how they restore temporal damage, are all strikingly similar. Both the intestinal and airways mucosae are patrolled by circulating lymphocytes, which serve as a direct immunological connection between the two organs, as these cells do not stay put. Any exposure to specific antigens is sensed by local dendritic cells, which shape future immune responses such as a T-helper cell type 1 (Th1) vs. a Th2 response and produces a longstanding impact on health and disease, in which the respective microbiomes play a key role. The crosstalk by which these organs ‘communicate’ is referred to as the “gut–lung axis”.

Most of the interorgan communications between the gut and the lung occur via the bloodstream. Immune responses that exert an interorgan effect include antibody-activated T-lymphocytes that respond to the local presence of pathogens and toxins, as well as anti-inflammatory cytokines (glucocorticoids) that are produced to limit an inflammatory reaction to only the necessary level [32]. Glucocorticoids (GCs) generally have anti-inflammatory functions (the anti-inflammatory drug Dexamethasone is a synthetic glucocorticoid). GCs are not only produced by the adrenal glands but also in smaller amounts by the epithelium of the gut and lung, where they downregulate local immune cell responses to avoid overreaction following a trigger. When pro-inflammatory tumor necrosis factor (TNF) is produced in the gut, it simultaneously stimulates the production of GCs, thus initiating the ‘brake’ that ensures a balanced inflammatory response [32]. Both the immunological defenses and the anti-inflammatory ‘brakes’ reach out beyond a locally infected area and enable the crosstalk between gut and lung. In addition, SCFAs produced in the gut reach the lungs via the bloodstream, where they can improve the health of lung cells just like they do in the gut. SCFAs are bound to G protein-coupled receptors (GPVCRs) which are expressed on various cell types; GPCRs are also coupled to signal transduction systems, which explains how SCFAs can have multiple effects, depending on tissues and cell types. This is treated in more depth elsewhere [33].

**Figure 1:**
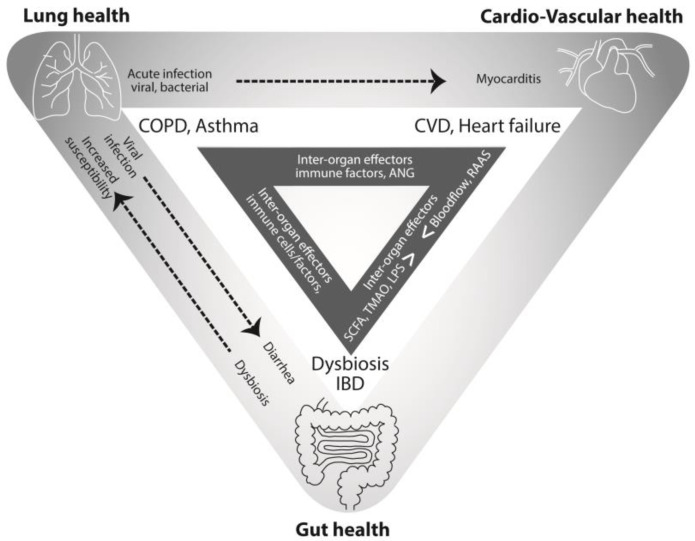
The interplay of lung health, cardiovascular health, and gut health. The dotted arrows indicate interorgan effects of local acute infections. The white triangle shows the main chronic conditions and the dark triangle lists some of the key effectors that enable communication.

Obviously, both the gut and the lung can be infected by pathogens, and an infection in the one can affect the health of the other. The microbial communities residing in the intestinal tract can shift as a result of a respiratory illness, and likewise, changes in the gut microbiome can affect the susceptibility of the lungs towards infection. Viral and bacterial respiratory infections can result in dysbiosis and intestinal symptoms [34]. As an example of effects in the opposite direction, the incidence or severity of airway infections is influenced by the gut microbiome [35]. This is indicated by the dotted arrows in Figure 1.

As stated above, the presence of pathogens is noticed by immune cells in either organ. Locally, this recognition is mainly the result of pathogen-associated molecular patterns (PAMPs) binding to pattern recognition receptors (PPRs). These PPRs, such as the Toll-like receptors (TLRs), act as the main ‘scouts’ that initiate an innate immune response if a pathogen is detected. This powerful branch of the immune system recognizes conserved motifs in macromolecules such as RNA or LPS, but it also responds to signals released by necrotic host cells; after all, infection is accompanied by cell damage, and even in the absence of a pathogen, damaged cells can be harmful and need to be eliminated. The TLRs in the gut notice the presence of pathogens and commensals alike, but they can differentiate between PAMPs or other danger-associated molecular patterns (DAMPs) and the harmless microbial MAMPs. When pathogens are thus recognized, the adaptive immune response is activated to initiate the production of pathogen-specific antibodies, including the IgA that is responsible for mucosal immunity.

The interconnections between the gut and the lungs have been investigated in detail in mice whose gut microbiota is altered by antibiotics. This treatment results in increased susceptibility for respiratory infections such as the influenza virus. Further, abiotic mice artificially lacking a gut microbiota have macrophages in their alveoli with changed transcriptomes that are less able to phagocytose bacteria, indicating the gut microflora is essential for correct macrophage function in the lungs. The interplay between the lungs and the gut is particularly important at both ends of the age spectrum: a diverse gut microbiome at an early age, for instance as a result of growing up on a farm, is protective of allergic asthma [36], while the common condition of dysbiosis in aging populations (associated with limited diet diversity) has been found to correlate to increased sensitivity to lung infections [37].

## 4. Connections between the Lung and the Heart

A number of acute viral and bacterial lung infections can result in myocarditis, and viral airway infections are among the more common causes of this condition. Some viruses can infect the heart (as some herpesvirus or enterovirus species do), while others (e.g., influenzavirus) induce myocarditis as a result of an overreactive immune response [38]. In their review, Favere et al. discuss in detail the role of TLRs in the onset of viral myocarditis. Most of the virus species that result in myocarditis are quite common, but animal experiments suggest genetic predisposition may provide a trigger to this potentially serious complication.

**Figure 2:**
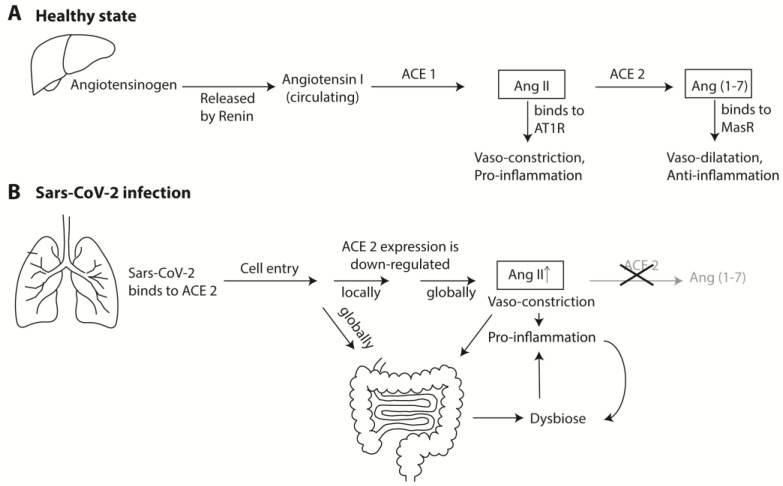
The roles of ACE1 and ACE2 as part of the Renin–Angiotensin System (RAS) in health and during SARS-CoV-2 infection. Panel **A** shows the production of Ang II and Ang(1-7) from angiotensinogen after it is produced in the liver. Their antagonistic effects on blood pressure and inflammation are shown. Alternative routes from Angiotensin 1 to give Ang II are not shown, and alternative components, receptors, and pathways are omitted for simplicity. Panel **B** summarizes the pro-inflammatory effect of ACE2 repression during SARS-CoV-2 infection. The gut increases the proinflammatory state with positive feedback loops.

Another mechanism lies behind the link between SARS-CoV-2 infection of the lungs and accompanying cardiac complications. SARS-CoV-2 primarily infects the upper airways, where its main docking receptor for cell entry is ACE2 (other proteins are required as well, which for simplicity is ignored here). ACE2 is a transmembrane protein expressed in many tissues. It has important exopeptidase enzymatic activity on peptides that modulate an array of critical pathways. It is well known as a key element of the renin–angiotensin system (RAS), which regulates blood pressure and fluid homeostasis. The main substrate for ACE2 is angiotensin II (Ang II), an eight amino acids-long peptide, which ACE2 shortens to the 7-mer Ang(1-7) (Figure 2, panel A). Ang II functions as a vasoconstrictor, whereas the ACE2 product Ang(1-7) is a vasodilator. Its precursor, angiotensinogen, is produced as a preproprotein in the liver and released as a decapeptide (angiotensin I) by the activity of renal renin, after which it is cleaved into the active octamer Ang II by the peptidase ACE1 (traditionally called just ACE). The octamer Ang II circulates in the bloodstream where it can, if needed, serve as a substrate for ACE2, which is expressed by many tissues including the vascular epithelium (minor amounts of functionally active ACE2 are also circulating as a result of ‘shedding’, carried out by yet another type of protease).

After ACE2 has converted Ang II into Ang(1-7), this peptide is now able to bind to the Mas receptor (MasR) on arteriole smooth muscle cells, resulting in vasorelaxation and a drop in blood pressure [39]. Thus, in broad strokes, high ACE2 activity lowers blood pressure, whereas low ACE2 activity increases blood pressure. ACE1 and ACE2 are responsible for the balance between the two counteracting hormones, Ang II and Ang(1-7) (Figure 1). In reality, far more enzymes and receptors are involved that are omitted here for clarity. The reader is directed to other reviews for more detailed information [39,40].

A similar counteractivity of Ang II and Ang(1-7) is observed for their effect on inflammation: generally speaking, Ang II is pro-inflammatory and Ang(1-7) is anti-inflammatory (again, with far more factors and effects involved than can be discussed here). Both ACE1 and ACE2 enzymes are involved in local (organ-specific) regulation of cell growth and proliferation, inflammation, and cytokine production, with mostly opposing effects [41]. ACE2 also has receptor properties (via its transmembrane and cytoplasmic tail interactions), as well as regulatory functions as a heterodimer partner in such important functions as transmembrane amino acid transportation by pairing with transporters. As such, it has a role in IBD [42].

ACE2 is expressed at higher levels on the apical surfaces of specific types of epithelial cells of the human upper and lower respiratory tract, where it can function as a receptor for respiratory viruses, including the common and less pathogenic coronaviruses. This was first demonstrated for the highly pathogenic SARS (now called SARS-CoV-1) and MERS [43,44]. The causative of COVID-19, SARS-CoV-2, avidly binds to ACE2 by means of its spike protein on a one–to–one molecular basis. The amount of available ACE2 molecules on the apical surface of the lung epithelium by far exceeds the number of virus particles that reach them, so that, at first, binding of relatively few virus particles will not directly affect the number of available ACE2 molecules. However, as soon as SARS-CoV-2 has entered a cell (for which additional enzymes are required), the expression of ACE2, in terms of its de novo production, its transmembrane insertion, and its transportation to the cell surface, is downregulated by the cellular responses to specific viral proteins. This not only happens in the infected cell, but also in the surrounding cells via antiviral paracrine signaling. This is how the local tissue attempts to limit the number of docking points for additional incoming virus particles and to restrict the eventual local spread of virus escaping the infected cell. At the same time, the amount of vasorelaxing and anti-inflammatory ACE2 functions are decreased, so that ACE1 counterfunctions can exert its vasoconstrictive and proinflammatory action. In this manner, the damage by the virus is isolated to the site of infection, while sequentially local and then regional innate immune response pathways are initiated (Figure 2, panel B).

Although much attention has been given to ACE2 expression in the respiratory tract, it is expressed at log-order higher levels in the intestinal system and gallbladder [45]. It has been demonstrated that, at least in mice, a deficiency of ACE2 causes inflammation, resulting in harmful alterations in the gut microbiome [46], which may be why gastrointestinal symptoms often accompany even mild cases of COVID-19. In the gut, ACE2 exopeptidase activity controls the local production of pro-inflammatory peptides by the gut epithelia and neuroendocrine cells; in addition, it enables the release of antimicrobial peptides by immune cells that combine to affect the daily healthy composition of the microbiome [47]. ACE2 also regulates immune responses via altering the regulation of gut tryptophan uptake by amino acid transporters. In summary, ACE2 is a key regulator of amino acid homeostasis, innate immunity, and gut (and probably lung) microbial ecology, in addition to being a key factor of RAS.

## 5. Learning from a Diseased State

The previous section on CVD demonstrates that the study of a diseased state can lead to insights on health, too. This applies to the chronic diseased states of both the gut and the lung. Chronic inflammatory bowel diseases (IBD), such as Crohn’s disease and ulcerative colitis, and chronic obstructive pulmonary disease (COPD) in the lungs, are associated with persistent local dysbiosis and have similar underlying mechanisms and risk factors. Both chronic disorders are associated with smoking and with certain genetic predispositions.

The chronic diseased state of the gut as in IBD, which is almost always accompanied by long-term microbiome dysbiosis, results in an increased prevalence of COPD, and vice versa [34], and a link to eczema is also apparent. As previously noted, IBD can be improved by SCFA supplementation. Both oral uptake of dietary fiber and of SCFA can contribute to airway homeostasis, as was demonstrated in mouse experiments [32]. In this context, it is no surprise that an association also exists between IBD and CVD; a cohort study in Taiwan indicated 27.5 times higher risk for peripheral arterial disease (PAD) in severe IBD patients [48].

Disruption of the gut microbiota composition (due to internal or external factors) can reduce the self-limiting capacity of the immune system and this can lead to chronic inflammatory conditions, not only of the gut itself but also by generating COPD and asthma [33]. Indeed, differences in the gut microbiome composition between asthma patients and controls have been demonstrated [49]. External factors that may disrupt the gut microbiota composition include diet, certain toxins and pathogens, and medications. Any of these triggers can cause dysbiosis, which may result in undesirable effects of the gut immunity that lead to local and systemic inflammation.

Dysbiosis in the gut has also been associated with obesity and diabetes, and obese individuals have an elevated pro-inflammatory state and a higher incidence of asthma, while weight loss (for instance by bariatric surgery) can reduce asthma [50,51]. The lung epithelium controls the local immune activities of IgA antibodies, defensins, and lysozymes, and regulates their production via cytokines that also stimulate a Th2-type inflammation, which in turn is implicated in the development of asthma [49]. The gut microbiome influences the Th2-immune response, and its composition may thus affect the onset of asthma, although the condition is thought to mainly originate from inhalation.

## 6. What Activities Would an Ideal Probiotic Need to Have?

So far, correlations between various health or disease states and gut microbiome observations have been mentioned and mechanisms of interorgan crosstalk have briefly been outlined. The larger question that remains to be answered is, can the gut microbiome be manipulated in such a way that the health status of an individual can be improved? This is what probiotics are considered to do.

The first thing to notice in this context is that the body does not differentiate between nonpathogenic gut flora and probiotic bacteria. The presence of commensals or transiently passing bacterial species in the gut will be ‘noticed’, via the host–microbe interactions discussed above, but as long as they do no harm (expressing PAMPs and producing local cell damage that raises alarms) these bacteria will virtually be left alone, to compete which each other for nutrients and a space to live, until they are being replaced by other species. The second thing to notice is that most bacterial species that have been investigated for their probiotic capacities are not only normal inhabitants of the gut but are also typically used in food fermentation. The choice for these is partly historical (the first probiotic activity was attributed to the bacteria present in unpasteurized yogurt) and partly practical (starter cultures are considered to be safe, making them easy candidates to apply as probiotics), but there is no guarantee that we have chosen the optimal species for investigations of probiotic activity. Indeed, over the past decade many review papers have discussed the absolute requirement of safety, but far fewer have addressed or assessed functionality, possibly because that goal is so much harder to reach. The other requirement much discussed in the literature is that probiotic bacteria should reach the gut alive, to take up residence and multiply there, i.e., ‘colonize’ for a nondefined period of time (mainly because that is part of the generally accepted definition of probiotics), though whether long-term colonization is desired or should be avoided remains disputed. In light of their proposed actions, it is difficult to imagine how dead bacteria can still have a persisting positive effect on health, which has nevertheless been proposed in the past [52]. Lastly, probiotic bacteria are typically industrially produced as monocultures, which ensures quality control and culture stability, although some commercial products contain mixtures of strains or species. However, some of the more important beneficial bacterial species of the gut are difficult to propagate in monocultures, including some of the main butyrate producers [25]. These can, however, propagate in mixed cultures, with increased butyrate production as a result [53,54]. Regulatory restrictions limit the use of mixed culture production, but maybe it is time to review such regulations, as the beneficial potential of combination cocktails of probiotic strains and species is evident.

Should we instead of the usual lactic acid bacteria choose bacterial species that are already commonly found in the gut of healthy individuals for probiotic use? Even if a bacterial species is commonly found in a human gut, there is no guarantee that every individual will be colonized to equal levels by that species, nor benefit in the same way. *E. coli* is again a key example: it is neither found in large quantities in the gut, nor is every individual easily colonized by it [55]. Another example of a less familiar Gram-negative bacterial species that is associated with healthy people but missing or present in very low abundance in people with obesity, diabetes, cardiometabolic diseases, and low-grade inflammation is *Akkermansia muciniphila*, which colonizes the intestinal mucosal layer and is proposed as a probiotic candidate [56]. As an example of a less frequently recognized Gram-positive species, an *Enterococcus faecalis* strain has been shown to be effective to prevent rhinosinusitis in children [57]. Further, as the examples discussed here illustrate, there are a wide variety of medical conditions correlating with intestinal dysbiosis, but it would be naive to assume that all of these can be improved with one type of bacterial probiotic in all affected individuals.

**Figure 3:**
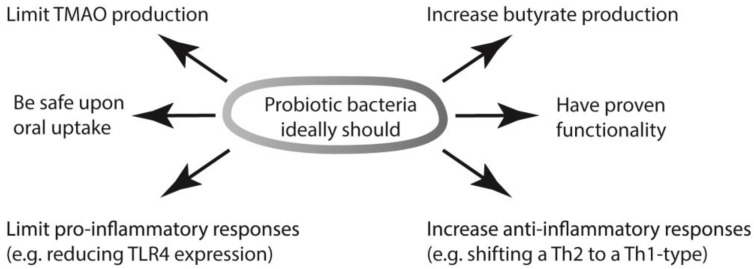
The required activities of ideal probiotic bacteria can now be proposed based on mechanistic understandings so that they can benefit the health of organs beyond the gut.

The variation in the microbiomes of individuals is extensive and varies over time, and even between the upper and the lower gut of a single person, which makes an educated guess for a broadly active probiotic bacterial strain even more difficult. However, a few general observations have repeatedly been mentioned here that may provide a basis: those species that produce SCFA, in particular butyrate, seem to be beneficial in multiple ways. The beneficial activities of SCFA even exceed those already mentioned, and also include elevation of intestinal IgA production, activation of T-reg cell differentiation, and regulation of epigenetic changes in immune cells [58]. In addition, bacteria that activate anti-inflammatory processes and limit pro-inflammatory actions can assist in tempering the immune response of a diseased atopic state. Lastly, bacteria that lack TMAO reductase or that reduce the numbers of other bacteria that do express this enzyme, would limit TMAO production, and this should be highly beneficial. These functions, summarized in Figure 3, are partly different from the mechanistic explanations frequently put forward to explain the health benefits of probiotic bacteria. Those traditionally considered probiotic functions include competition with and removal of pathogens. Such antagonism was the original criterion by which Alfred Nissle isolated *E. coli* Nissle 1917 [55]. Other modes include inhibiting pathogens from adhering to the mucosal surface, lactose consumption and lactic acid production, and antioxidant activity [59,60].

The importance of butyrate production has been long established [61], but the focus on intestinal disorders may have restricted an assessment of its full potential. That probiotics can assist in the prevention of respiratory infections is now well established and this was confirmed in meta-analyses [62,63]. The mechanisms of immunomodulation are now better understood [64]; instead of a generalization such as ‘stimulation of the immune system’, it is now possible to describe systemic effects more accurately. The scene is now set to search for such activities (as summarized in Figure 3) in bacteria that can colonize the gut, thereby extending investigations well beyond the known probiotic archetypes. The search for new probiotic candidates must ideally be based on randomized clinical studies, just like those that are required for drugs and vaccines, as has been stated before [52]. In some parts of the world, including the EU, these requirements are already in place to register novel products that claim health benefits. However, clinical trials are costly, and this may be a reason why so few efforts for development of probiotics have started by assessment of safety in combination with functionality (the two targets that, together with appropriate dose, are essentially addressed in clinical trials), instead of testing known safe bacteria for any beneficial effect that they might have. If functionality must be proven, the discussion of whether probiotic bacteria must reach the gut alive and colonize there will most likely finally come to a definite conclusion, as without that capacity to colonize, it will be hard to provide such proof.

Examples of randomized clinical trials performed with the usual suspects for probiotics are more abundant, and a large number of meta-analyses have been published that assessed their effects on conditions of relevance here. These are summarized in Table 1. Since meta-analyses combine the results of past studies in an accumulative manner, only those published since 2016 were included in the table. Far more meta-analyses did find an effect of probiotics, for a variety of conditions, compared to those that did not find significant overall effects. Any subgroup analyses that may have been reported are not considered here. There are noted limitations for this sort of research: the effects scored in meta-analyses are often minor, study design and quality of the investigated trials vary considerably, and trials are often highly heterogeneous, with multiple probiotic species, strains and doses being tested. Nevertheless, we conclude that an overwhelming number of clinical trials have now been published that report functionality of probiotics for the conditions of interest here.

## 7. Probiotics and COVID-19

It would be naive to think that probiotics can completely prevent SARS-CoV-2 infection or COVID-19 symptoms, but since a healthy gut microbiome has obvious health benefits to multiple organs, probiotics have been proposed as one of the multiple strategies to ameliorate SARS-CoV-2 infections [6,114,115,116,117,118].

Early observations from China have suggested that some COVID-19 patients suffered from dysbiosis with fewer *Lactococcus* and *Bifidobacterium* species in their gut. Infection with SARS-CoV-2 not only downregulates ACE2 expression in the lungs but also in the gut [45], and since ACE2 tightly controls the local production of antimicrobial peptides in the gut, this may have direct effects on the microbiome. Another explanation that has been put forward for gut dysbiosis is virus replication in the gut epithelial tissue. However, although prolonged viral genomic RNA shedding can sometimes be detected in stools, active replication in the gut does not take place on a large scale.

Immune modulation of the gut microbiome in response to an infection of the respiratory tract is also a possibility. Many of the symptoms related to COVID-19 are due to an overactive immune response, resulting in a potentially lethal cytokine storm and acute respiratory distress syndrome (ARDS) [118]. Interestingly, bacteria associated with inflammation, such as *Blautia* and *Ruminococcus* species, were more abundant in the gut microbiota of symptomatic COVID-19 patients, as reviewed by others [119].

Such observations suggest that individuals might reduce the occurrence and severity of COVID-19 symptoms when their gut contains sufficient levels and diversity of immune-regulating probiotic bacteria. Probiotics with anti-inflammatory activity might significantly reduce the risk of a cytokine storm [120], while a range of inflammatory mediators responsible for ARDS can also be decreased by probiotics [116]. Some probiotics have been described that reduce ACE1 expression [121], which would contribute to an anti-inflammatory response via the RAS system, as in Figure 2. It has further been suggested that certain bacterial species can regulate ACE2 expression in the gut, with possible beneficial downstream effects on the lungs via RAS [58,122], although it remains to be demonstrated if this is feasible by the administration of particular probiotics. The production of butyrate would obviously assist in repairing damage to the gut and the lung epithelial linings. The ability of some probiotics to increase the steadystate levels of type I interferons, which have systemic antiviral activity, can also contribute [65]. Even as the pandemic is waning as a result of vaccination, probiotics may assist in this, as vaccine success can be enhanced by probiotics [58]. This seems counterintuitive, as most of the beneficial activity of probiotics seems to be anti-inflammatory, but a healthy well-balanced immune system responds more completely and correctly to vaccines, and the positive effect of probiotics on vaccination may be due in part to an adjuvant-like effect of their LPS [123].

Lastly, patients who only experienced relatively mild or no symptoms during the acute phase of a SARS-CoV-2 infection, can later develop a variety of symptoms for a prolonged period of time that are collectively described as ‘long COVID-19’ [124]. The true burden of this postinfectious sequel is not yet clear. Symptoms can last for months, but anecdotal evidence suggests that the condition can improve after receiving a COVID-19 vaccination. It appears that, at least in some individuals, dysfunctional checks and balances in the complex immune system can be reset by the right input, likely via immune regulation. In this context, it would be interesting to see if probiotics can assist in a faster recovery of long-COVID-19 patients.

## 8. Conclusions

For decades, probiotics have been suggested as solutions for a wide variety of medical conditions, thereby providing a solution for problems that kept changing, ranging from severe chronic diseases to a general and nondefined ‘improvements in health’. This has understandably resulted in a degree of well-deserved skepticism, in particular when activities in organs other than the gut were supposedly targeted. However, with a better understanding of the interconnectedness of organs, via chemokines, cytokines, neuroendocrine molecules, migrating immune cells, specific metabolites, and even the nervous system, it is now becoming clear that bacteria in the gut can indeed have far-reaching and systemic consequences, and that a healthy gut microbiome can contribute to the prevention, mitigation, and healing of a number of conditions. Novel insights have also resulted in a better-defined ‘wish list’ for an ideal probiotic. It is unlikely that we have as yet identified the best candidates for the most beneficial bacteria for different conditions, as the usual candidates were selected for based on criteria other than their functional properties. Naturally, safety was the utmost important criterion, and industrially-used bacteria that were already considered safe upon oral intake were the logical first choice candidates. The research conducted with these probiotic bacteria has admittedly led to valuable insights. Now that we have started to better understand exactly how probiotic bacteria work, the next decade of research may result in more suitable and more potent candidates. At least it is no longer a mystery if and how gut bacteria can be beneficial for lung conditions or heart disease. If these novel proffered insights are used to guide the next phase of hypothesis-driven research, more breakthroughs can be expected.

## Figures and Tables

**Table 1:** Meta analyses published since 2016 assessing the activity of probiotics towards conditions related to the heart–lungs–gut triangle.

Target Condition	Reported Effects (1)	Nr of Trials Included [Reference]
Improving natural killer cell function	No significant effect	6 [65]
Oxidative stress	Improved antioxidant markers	16 [66]
Inflammatory markers	Reduced pro-inflammatory markers	42 [67]
Inflammation in elderly	No significant effect	10 [68]
Glycemic response, BMI, T2D	Improved glucose metabolism, reduced BMI	14 [69]
T2D	Reduced dyslipemia, improve metabolic control	32 [70]
T2D	Reduced insulin resistance	15 [71]
T2D	Improved oxidative stress biomarkers	13 [72]
T2D	Improved inflammatory markers	18 [73]
T2D	Improved inflammatory and oxidative stress markers	16 [74]
T2D	Improved blood lipid profile, reduced BP	10 [75]
T2D	Reduced insulin resistance	12 [76]
T2D	Reduced insulin resistance and blood lipid profile	12 [77]
T2D	Lowered fasting glucose levels	12 [78], 14 [79]
High BMI	Reduced waist circumference, no effect on BMI	19 [80]
High BMI	No significant effect	9 [81]
High BMI	Reduced total cholesterol, LDL	12 [82]
High BMI	Reduced body weight	15 [83]
High BMI (synbiotic treatment)	Reduced waist circumference and body weight	23 [84]
Obesity, T2D, NAFLD	Minor improvements of metabolic risk factors	15 [85]
NAFLD	Improved liver-specific markers	21 [86]
Not specified	Reduced LDL, total cholesterol	39 [87]
Lipidaemia	Reduced LDL, total cholesterol	19 [88], 21 [89], 15 [90], 12 [91]
Metabolic syndrome	No significant effect for most biomarkers, but reduced LDL	18 [92]
UC	Induced remission	14 [93], 3 [94]
Crohn’s Disease and UC	No significant effect	18 [95]
IBD	Improved symptoms	33 [96], 35 [97]
IBD	No significant effect	10 [98]
IBD	Induced remission	22 [99]
IBD (Constipation-dominant)	Increased stool frequency	17 [100]
IBD, side effect assessment	Increased abdominal pain	9 [101]
Crohn’s disease	No clear effect	2 [102]
IBS	Improved symptoms	14 [103], 5 [104], 22 [105]
Prevention of RTI	Reduced incidence of RTI	16 [62]
Pediatric RTIs	Reduced risk of RTI	23 [106]
Pediatric asthma, allergic rhinitis	No significant reduction	17 [107]
Pediatric asthma	No significant reduction	19 [108]
Allergic rhinitis	Reduced nasal and ocular symptom scores	5 [109]
Hypertension	Lowered systolic BP	7 [110]
CVD, cholestrol	Reduced total cholesterol	32 [111]
CVD	No significant effect	unclear [112]
Vaccination efficiency (influenzavirus)	Enhanced antibody titers	12 [113]

Abbreviations: BMI: body mass index; T2D: Type 2 diabetes; NAFLD: non-alcoholic fatty-liver disease; LDL: low-density lipoprotein; RTI: respiratory tract infections; UC ulcerative colitis; IBD: inflammatory bowel disease; IBS: irritable bowel syndrome; BP: blood pressure. (1) Any reported effects of subgroup analyses are not included in the table.
